# Formation of S-Bearing Complex Organic Molecules in Interstellar Clouds via Ice Reactions with C_2_H_2_, HS, and Atomic H

**DOI:** 10.1021/acsearthspacechem.4c00150

**Published:** 2024-07-25

**Authors:** Julia C. Santos, Joan Enrique-Romero, Thanja Lamberts, Harold Linnartz, Ko-Ju Chuang

**Affiliations:** † Laboratory for Astrophysics, Leiden Observatory, Leiden University, PO Box 9513, 2300 RA Leiden, The Netherlands; ‡ Leiden Institute of Chemistry, Gorlaeus Laboratories, Leiden University, PO Box 9502, 2300 RA Leiden, The Netherlands; ⊥ Leiden Observatory, Leiden University, 2300 RA Leiden, The Netherlands

## Abstract

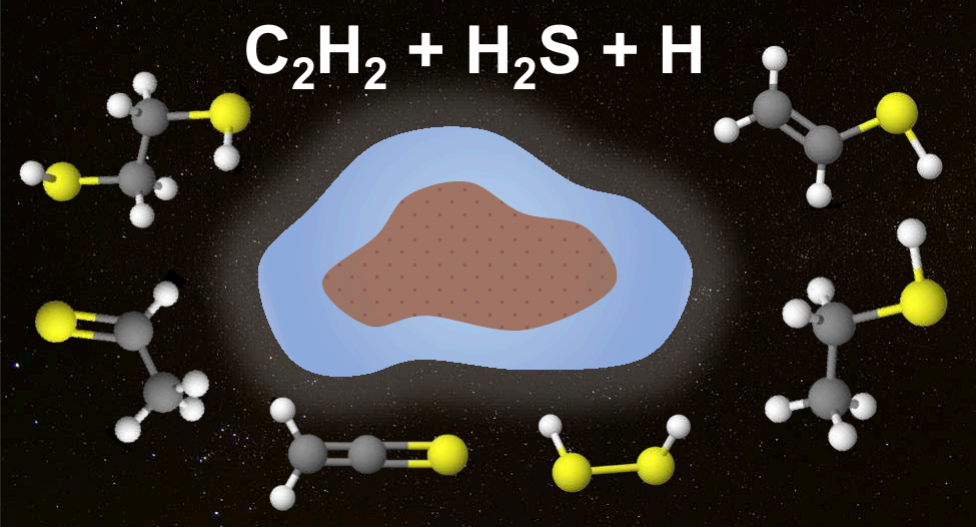

The chemical network governing interstellar sulfur has been the topic of unrelenting discussion for the past few decades due to the conspicuous discrepancy between its expected and observed abundances in different interstellar environments. More recently, the astronomical detections of CH_3_CH_2_SH and CH_2_CS highlighted the importance of interstellar formation routes for sulfur-bearing organic molecules with two carbon atoms. In this work, we perform a laboratory investigation of the solid-state chemistry resulting from the interaction between C_2_H_2_ molecules and SH radicals—both thought to be present in interstellar icy mantles—at 10 K. Reflection absorption infrared spectroscopy and quadrupole mass spectrometry combined with temperature-programmed desorption experiments are employed as analytical techniques. We confirm that SH radicals can kick-start a sulfur reaction network under interstellar cloud conditions and identify at least six sulfurated products: CH_3_CH_2_SH, CH_2_CHSH, HSCH_2_CH_2_SH, H_2_S_2_, and tentatively CH_3_CHS and CH_2_CS. Complementarily, we utilize computational calculations to pinpoint the reaction routes that play a role in the chemical network behind our experimental results. The main sulfur-bearing organic molecule formed under our experimental conditions is CH_3_CH_2_SH, and its formation yield increases with the ratios of H to other reactants. It serves as a sink to the sulfur budget within the network, being formed at the expense of the other unsaturated products. The astrophysical implications of the chemical network proposed here are discussed.

## Introduction

Over 300 molecules have been detected in the interstellar and circumstellar medium to date,^1^ with identifications increasing at a remarkably accelerating rate within the past decade.^2^ Among such detections, the so-called “complex organic molecules” (COMs, i.e., organic molecules with six or more atoms) are observed in various sources at different stages of star and planet formation—from prestellar cores to comets.^3−12^ Their formation routes have been extensively investigated in both gas and solid phases (see the reviews by Herbst,^9^ Linnartz et al.,^13^ and Öberg^14^). Radical-induced reactions can lead to the formation of a wide range of complex organic molecules even at temperatures as low as 10 K.^13,15−22^ Alternatively, interactions of closed-shell species with photons, electrons, or cosmic rays can also trigger chemical reactions on the ice. Investigating the different pathways to form interstellar molecules, and especially COMs, is therefore crucial to understanding the evolution of the chemical inventory of different sources.

Sulfur-bearing species, in particular, have been a longstanding issue in astrochemistry. In dense environments such as cold clouds, the (observable) gas-phase sulfur is severely depleted by up to 2 orders of magnitude compared to cosmic values,^23−27^ with the bulk of its content remaining largely unknown. In addition to the sulfur depletion problem, hydrogen sulfide (H_2_S) specifically also exhibits a mismatch between its predicted abundances—based on astrochemical models—and observations. It is formed very efficiently in ices via the successive hydrogenation of S atoms, producing the radical SH as an intermediate (see, e.g., Garrod et al.,^28^ Druard and Wakelam,^29^ Esplugues et al.,^30^ and Vidal et al.^31^):
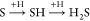
1

Indeed, H_2_S has been detected in the gas phase toward a range of different interstellar sources.^32−42^ It has also been observed in the comae of comets at the highest abundance amid all sulfur-bearing species.^39−41,43^ However, it has not been detected in interstellar ices so far, and estimated upper limits are only ≤0.7% with respect to water.^44^ This indicates that H_2_S ice must be subjected to effective destruction mechanisms, among which chemical desorption and (photo)chemical conversion seem to be particularly promising.^44−50^

A prominent loss channel involves the interaction of H atoms with solid H_2_S, resulting in the abstraction reaction
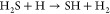
2which involves a barrier of ∼1500 K that can be overcome by quantum tunneling.^51^ This reaction has been shown to enrich the ice mantles with SH radicals, which in turn can kick-start sulfur-bearing ice chemistry at temperatures of relevance to molecular clouds.^50^ On a similar context, Laas and Caselli^52^ found that a significant portion of the missing sulfur reservoir would consist of simple organosulfur compounds (e.g., OCS, H_2_CS, CS_2_) locked up in the ices. Solid-state reactions leading to S-bearing molecules could thus play a significant role in unraveling the fate of interstellar sulfur.

Another important species for interstellar chemistry is the simplest alkyne, acetylene (C_2_H_2_). It has been observed with infrared instruments in the gas phase toward young stellar objects and envelopes of C-rich stars, as well as at fairly high abundances in the inner parts of protoplanetary disks and in comets.^53−60^ It is also suggested to be present in prestellar clouds.^61,62^ However, definitive detections in such environments require observations of gaseous molecules at submillimeter wavelengths, which are hindered for C_2_H_2_ due to its lack of a dipole moment. Since gas-phase formation routes cannot explain its observed abundances, sublimation from dust grains is usually suggested as a major source of gaseous C_2_H_2_.^55,63,64^ However, its main ice features at ∼3 and ∼13 μm overlap with H_2_O and silicate bands, making its detection quite challenging.^65,66^ This is particularly true for water-rich ices, as evinced by its high upper limit of 10% with respect to H_2_O.^65^

A top-down mechanism through the energetic processing of polycyclic aromatic hydrocarbons (PAHs) or bare carbonaceous grains has been shown experimentally to be particularly efficient in forming C_2_H_2_ (see, e.g., Jochims et al.,^67^ Le Page et al.,^68^ and West et al.^69^). Additionally, a bottom-up formation could be feasible through the diffusion and reaction of C atoms on dust grains yielding C_2_, and its subsequent hydrogenation.^70^ The widely detected C_2_H radicals (e.g., Padovani et al.,^71^ Sakai and Yamamoto,^72^ and Kastner et al.^73^) could also contribute to forming C_2_H_2_ upon adsorption onto ice grains followed by hydrogenation. Its gas-phase abundances in prestellar cores can however be lower than C_2_H_2_ counterparts detected toward hot cores by up to 1 order of magnitude,^55,71^ which might limit its role as a dominant C_2_H_2_ precursor. As the cloud becomes denser and the residence time of H atoms on the dust surfaces increases, C_2_H_2_ may be hydrogenated to form C_2_H_4_, C_2_H_6_, and the radicals in between.^74−76^

The reactive nature of the triple bond in C_2_H_2_ makes it a versatile precursor to form interstellar molecules with a carbon backbone (see, e.g., the routes proposed by Molpeceres and Rivilla^76^ and Charnley^77^). Indeed, laboratory investigations of its reaction with thermalized OH radicals and H atoms on interstellar-ice analogues resulted in the formation of a variety of O-bearing COMs, such as acetaldehyde (CH_3_CHO), vinyl alcohol (CH_2_CHOH), ketene (CH_2_CO), and ethanol (CH_3_CH_2_OH).^19^ Furthermore, energetic processing of C_2_H_2_ ices mixed with other interstellar-relevant compounds (e.g., H_2_O, CO, CO_2_, and NH_3_) has also been explored experimentally, probing efficient formation mechanisms to a wide range of O- and N-bearing molecules.^78−83^ Its reactivity with sulfur-bearing species, however, remains to be explored under interstellar cloud conditions.

In a recent computational study, Molpeceres and Rivilla^76^ suggested a general mechanism in which C_2_H_2_ molecules could provide the carbon backbone to form a series of different organic molecules by reacting with a range of open-shell species (e.g., H, OH, NH_2_) on interstellar-ice surfaces. Among other cases, they speculate that its interaction with H atoms followed by SH radicals could serve as a potential route to forming S-bearing COMs in the solid state, albeit without exploring it computationally. In the present study, we conduct an experimental investigation of the formation of S-bearing COMs via the interactions of C_2_H_2_ with H atoms and SH radicals. We also employ quantum-chemical calculations to elucidate the reaction network behind our laboratory results. In the Methods section, the experimental setup and computational methods are described. The main results are presented and discussed in the Results and Discussion, and their astrophysical implications are elaborated in the next section. In Conclusions, we summarize our main findings.

## Methods

### Experimental Methods

This work is executed with the ultrahigh-vacuum (UHV) setup SURFRESIDE^3^, which has been described in detail elsewhere.^84,85^ Here, only the relevant information is presented. A gold-plated copper substrate is mounted on the tip of a closed-cycle helium cryostat at the center of the main chamber, which operates at a base pressure of ∼5 × 10^–10^ mbar. The substrate temperature can be varied between 8 and 450 K using resistive heaters and is monitored by two silicon diode sensors with a relative accuracy of 0.5 K. Gases of H_2_S (Linde, purity 99.5%) and C_2_H_2_ (Linde, 5% in helium) are simultaneously admitted into the chamber through all-metal leak valves. Concomitantly, a hydrogen atom beam source (HABS^86^) generates H atoms that are subsequently cooled down by colliding with a nose-shaped quartz pipe before reaching the substrate. Ice is deposited at 10 K and monitored by Fourier-transform reflection-absorption infrared spectroscopy (FT-RAIRS), with 1 cm^–1^-resolution spectra acquired in the range of 700–4000 cm^–1^. After deposition, the sample is heated at a ramping rate of 5 K min^–1^ during temperature-programmed desorption experiments (TPD). The sublimated ice species are immediately ionized by electron impact with an energy of 70 eV and are recorded by a quadrupole mass spectrometer (QMS), while the solid phase is concurrently monitored by RAIRS.

To quantify the abundances of products, two approaches are adopted. In the case of infrared spectroscopy, the IR integrated absorbance (∫Abs(ν)d*ν*) of the species in the ice can be converted to absolute abundance using a modified Beer–Lambert law
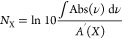
3where *N*_X_ is the column density in molecules cm^–2^ and *A*′(X) is the apparent absorption band strength in cm molecule^–1^ of a given species. For H_2_S, we utilize  cm molecule^–1^, as measured for our reflection-mode IR settings using the laser-interference technique.^50^ The band strength of C_2_H_2_ is derived by multiplying the *A* value in transmission mode reported by Hudson et al.^87^ ( cm molecule^–1^) by a transmission-to-reflection conversion factor of 3.2 measured with the same experimental setup (see Santos et al.^50^ for the case of H_2_S, that was later combined with the value for CO to derive the averaged conversion factor).

Due to the higher sensitivity of the QMS compared to the RAIRS employed in this work, some of the chemical species formed in our experiments were only detectable by the former technique. In order to quantify their relative abundances, column density ratios (*N*_X_/*N*_Y_) can be derived from the QMS data by the expression:^88^
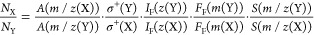
4where *A*(*m*/*z*) is the integrated desorption signal of a given mass fragment, *F*_F_(*m*/*z*) is its fragmentation fraction, and *S*(*m*/*z*) is the corresponding sensitivity of the QMS. Moreover, σ^+^ denotes the molecule’s electronic ionization cross-section and *I*_F_(*z*) is the fraction of ions with charge *z* (here corresponding to unity). The parameters employed for each of the products found in this work are summarized in Table 1. Given the overall lack of experimental values, the ionization cross sections are estimated based on the molecule’s polarizability volume (α) by the empirical correlation^89,90^
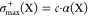
5where X denotes a given species and *c* is a correlation constant of 1.48 Å^–1^. It is expected that, for organic species, σ^+^(X) at 70 eV does not vary significantly (<5%) from the maximum ionization cross section (σ_max_^+^).^93,94^ When available, fragmentation fractions are derived from the NIST.^125^ Otherwise they are estimated based on our QMS measurements. In the case of CH_2_CHSH, isolating its peak during TPD is quite challenging (see Results and Discussion). Thus, we assume the same fragmentation fraction as its fully hydrogenated counterpart, CH_3_CH_2_SH. The polarizability values are obtained from CCCBDB.^126^ Given sulfur’s natural isotopic abundances, the contribution from ^34^S-bearing species to the mass signals would be ∼22 times weaker than the dominant ^32^S-bearing counterparts and therefore are not further considered.

**Table 1:** Parameters Used to Quantify the Products’ Relative Abundances with the QMS

species	α (Å^3^)^a^	*F*_F_ (*m*/*z*)^b^	*S* (*m*/*z*)^b^^,^^c^
CH_3_CHS	7.617^d^	0.598^g^	0.1
CH_2_CHSH	7.578^e^	0.200^h^	0.1
CH_3_CH_2_SH	7.38^f^	0.200^i^	0.09
H_2_S_2_	6.828^d^	0.330^g^	0.08
HSCH_2_CH_2_SH	10.503^d^	0.164^i^	0.1

a CCCBDB.

b Values are given for the molecular ions.

c Chuang.^91^

d Derived by group additivity methods.

e Computed with the B97D3/daug-cc-pVTZ level of theory.

f Gussoni et al.^92^

g Derived in this work.

h Assuming the same as CH_3_CH_2_SH.

i NIST.

The experiments performed in this work are summarized in Table 2. Both molecule fluxes, as well as the H atom flux, have an estimated relative error of ≲5%. As a control experiment, pure CH_3_CH_2_SH (Sigma-Aldrich, purity 97%) ice is grown through vapor deposition. Previous to dosing, it is purified by a series of freeze–pump–thaw cycles. The other identified products are not commercially available, and therefore, standard samples cannot be obtained. The instrumental uncertainties in the integrated QMS signals are derived from the corresponding integrated spectral noise for the same bandwidth.

**Table 2:** Overview of the Performed Experiments

experiment	label	C_2_H_2_ flux (cm^–2^ s^–1^)	H_2_S flux (cm^–2^ s^–1^)	H flux (cm^–2^ s^–1^)	*C_2_H_2_:H_2_S:H*	time (min)
C_2_H_2_ + H_2_S + H	1	5.0 × 10^11^	5.0 × 10^11^	1.0 × 10^13^	1:1:20	360
C_2_H_2_ + H_2_S + H	2	7.5 × 10^11^	3.7 × 10^12^	7.5 × 10^12^	1:5:10	80
C_2_H_2_ + H_2_S + H	3	8.0 × 10^11^	8.0 × 10^12^	4.0 × 10^12^	1:1:5	60
C_2_H_2_ + H_2_S + H	4	6.0 × 10^11^	6.0 × 10^11^	6.0 × 10^12^	1:1:10	240
C_2_H_2_ + H_2_S + H	5	2.0 × 10^11^	2.0 × 10^11^	1.0 × 10^13^	0.4:0.4:2	360
C_2_H_2_ + H	6	6.0 × 10^11^		6.0 × 10^12^	1:0:10	240

experiment	label	CH_3_CH_2_SH flux (cm^–2^ s^–1^)	time (min)
CH_3_CH_2_SH	7	7.0 × 10^12^	60

### Computational Methods

We complemented our experimental results with computational chemical simulations for selected cases. Specifically, we have employed ORCA 5.0.4^95^ to run density functional theory (DFT) calculations. The density functional of choice is M062X,^96^ combined with the def2-TZVP basis set. All the DFT calculations were done under the unrestricted formalism, and the options VERYTIGHTSCF and NORI were used. The broken symmetry approximation was used in all open shell singlet systems (i.e., radical–radical reactions). We made sure M062X is an appropriate method by comparing its performance with CCSD(T)-F12/AUG-CC-PVTZ calculations in radical-molecule reactions. For these coupled cluster calculations we used Molpro.^97−99^ The results can be found in Computational Results and Chemical Network.

## Results and Discussion

**Figure 1:**
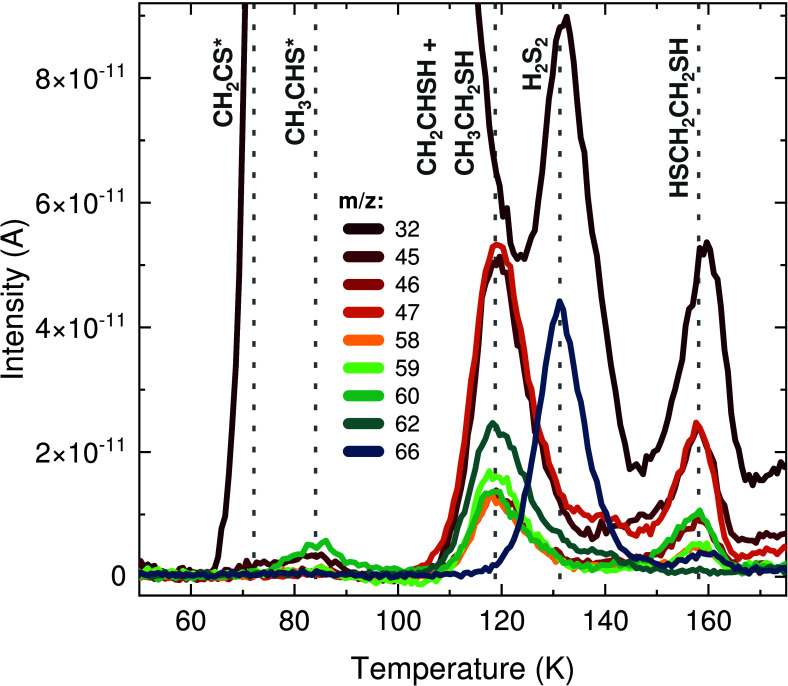
Relevant QMS signals recorded during a TPD experiment after codepositon of C_2_H_2_:H_2_S:H (1:1:20) at 10 K for 6 h. The dotted lines mark the desorption peak of each band, and their assignments are denoted in gray. The asterisks indicate tentative identification.

**Figure 2:**
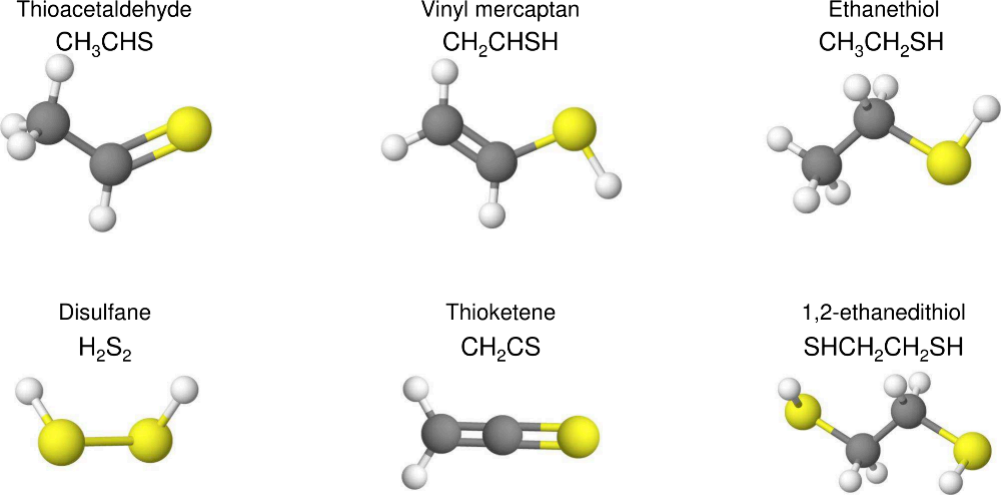
Representative structures of the six S-bearing products identified in the experiments with C_2_H_2_ + H_2_S + H. These are provided for visualization purposes and may not depict the accurate minimum-energy geometries of the species formed in the experiments.

In Figure 1, the selected signals of *m*/*z* = 32, 45, 46, 47, 58, 59, 60, 62, and 66 recorded during TPD after the codeposition of C_2_H_2_, H_2_S, and H (1:1:20) for 6 h (experiment 1) are shown. The deposition experiment results in (at least) six reaction products as revealed by a prolific series of desorption peaks at, in decreasing order of signal intensity, ∼119, 131, ∼158, ∼84, and ∼72 K. These bands are identified as six sulfur-bearing molecules: respectively, ethanethiol (CH_3_CH_2_SH), vinyl mercaptan (CH_2_CHSH), disulfane (H_2_S_2_), 1,2-ethanedithiol (HSCH_2_CH_2_SH), thioacetaldehyde (CH_3_CHS), and thioketene (CH_2_CS). Representative structures of the identified products are shown in Figure 2 as obtained from the MolView application.^127^ In the following subsections, the assignments of the desorption bands depicted in Figure 1 are discussed in detail.

### CH_2_CHSH and CH_3_CH_2_SH

**Figure 3:**
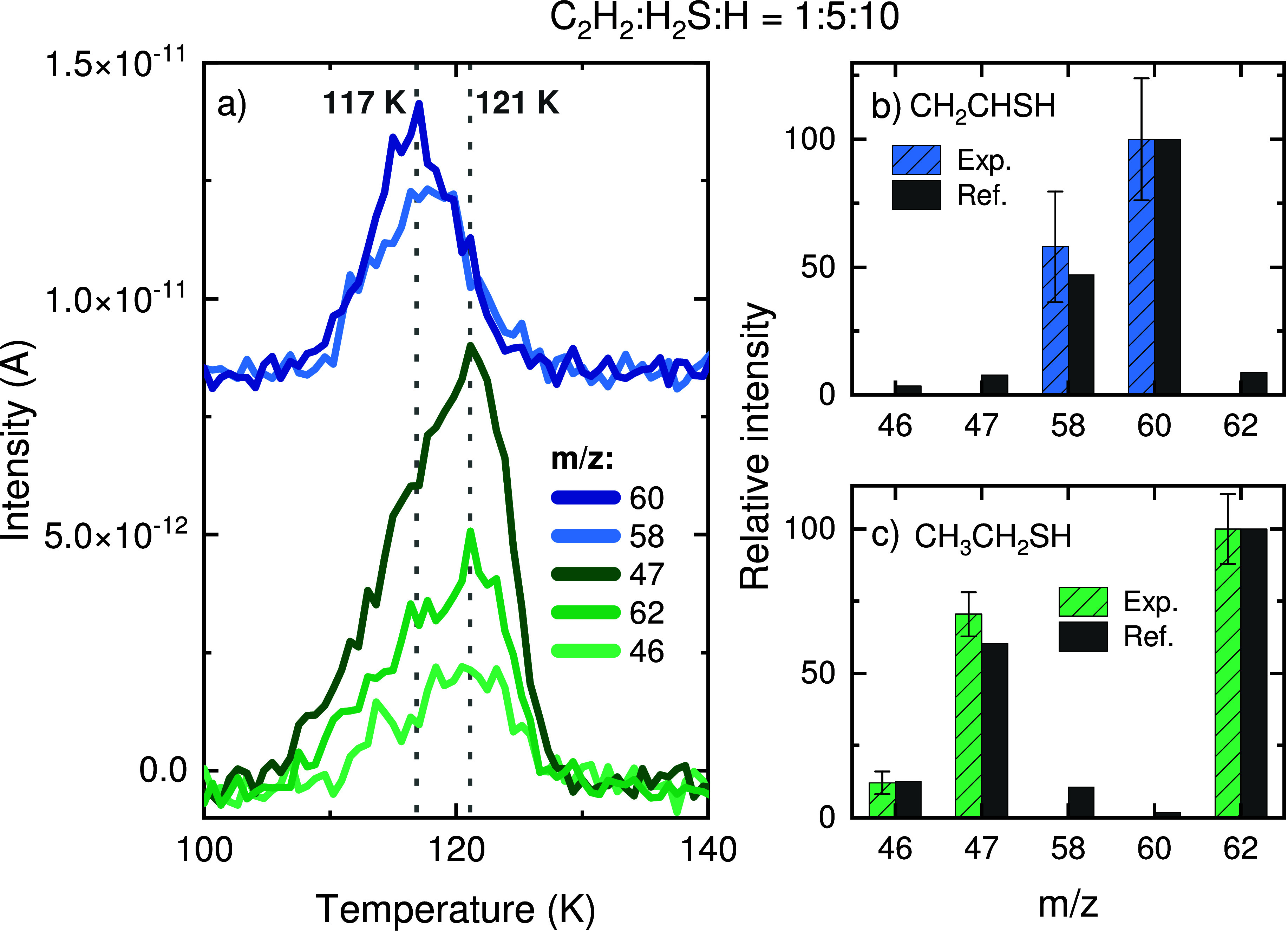
Assignment of the TPD-QMS peaks at ∼117 and ∼121 K as vinyl mercaptan (CH_2_CHSH) and ethanethiol (CH_3_CH_2_SH), respectively. The deposition is performed with a flux ratio of C_2_H_2_:H_2_S:H = 1:5:10. (a) QMS signals showing two desorption peaks highlighted by the dotted gray lines. (b) Mass fragmentation pattern of the selected signals for the peak at ∼117 K corrected for the sensitivity of the QMS. The standard for CH_2_CHSH is shown for comparison.^100^ (c) Same as (b) but for the peak at ∼121 K in comparison with CH_3_CH_2_SH (standard measured in this work).

The TPD of experiment 2 (C_2_H_2_:H_2_S:H = 1:5:10) is shown in Figure 3a. In contrast to experiment 1, for this mixing ratio two desorption peaks—at ∼117 and ∼121 K—are clearly distinguishable, revealing that this band actually consists of two products. The first one is evinced by a peak in the signals for *m*/*z* = 58 and 60, whereas the second one is characterized by *m*/*z* = 46, 47, and 62. Given the elemental composition of the ice (i.e., C, S, and H atoms), these mass signals are consistent with the desorption of two sulfur-bearing organic molecules with general formula C_2_H_*X*_S. From the peak signals of *m*/*z* = 60 and 62—the largest mass-to-charge ratios detected for each peak respectively—it is inferred that the product desorbing at ∼117 K is described by the formula C_2_H_4_S, and the one at ∼121 K is described by C_2_H_6_S. Indeed, it is expected that the lighter species with four hydrogen atoms would be more volatile than the fully saturated counterpart, albeit slightly. The contribution of the mass fragment *m*/*z* = 32 to these species cannot be measured since it is blended with the desorption profile of H_2_S at ∼85 K (see, e.g., Santos et al.^50^).

Given the proposed chemical route involving C_2_H_2_, SH radicals, and H atoms, the most plausible candidates to the ∼117 and ∼121 K peaks are respectively vinyl mercaptan (CH_2_CHSH) and ethanethiol (CH_3_CH_2_SH). These assignments are fortified by a comparison with the molecules’ standard fragmentation pattern, although deriving the mass fragments’ relative intensities is not trivial in this case. Since the full desorption bands of the two products still overlap considerably, the quantification of the contribution from each species to a given integrated mass signal is challenging. To minimize errors induced by contamination from the blended molecule, only fingerprint *m*/*z* signals uniquely associated with each species were considered in the product identification—i.e., mass fragments with a simultaneous relative intensity of ≥10% in both standards of CH_2_CHSH and CH_3_CH_2_SH were excluded from the analysis, leaving only the ones with minor contributions from the adjacent feature. Figure 3b,c shows the resulting fragmentation patterns of peaks ∼117 and ∼121 K, respectively, as well as the reference values for CH_2_CHSH and CH_3_CH_2_SH. The relative intensities of each peak match the references well, securing their assignments as CH_2_CHSH and CH_3_CH_2_SH. Furthermore, the pure CH_3_CH_2_SH ice standard exhibits peak desorption at 119 K, in line with its identification in the C_2_H_2_ + H_2_S + H experiments.

**Figure 4:**
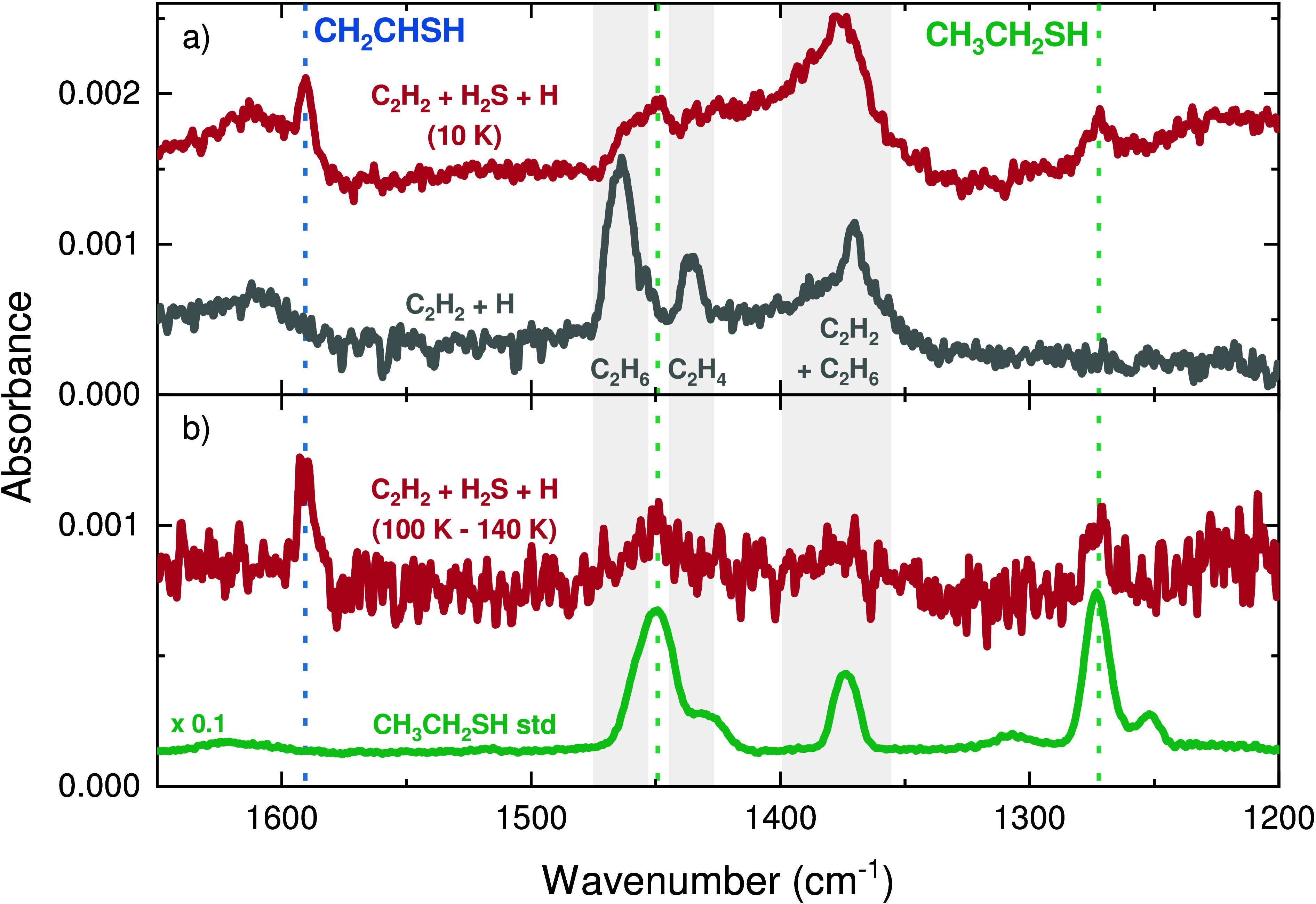
Infrared spectra utilized to assign CH_3_CH_2_SH and CH_2_CHSH. (a) Spectrum recorded after codeposition of a C_2_H_2_ + H_2_S + H ice (1:1:20; red), together with a C_2_H_2_ + H (1:10; gray) blank experiment. (b) Difference spectrum acquired during TPD between 100 and 140 K (red) for the C_2_H_2_ + H_2_S + H codeposition, together with a CH_3_CH_2_SH standard spectrum (green). All depositions were performed at 10 K, and the spectra are offset for clarity.

Infrared spectroscopy is used to further assist in the identification of the most abundant products. The red IR spectrum in Figure 4a was acquired after a 6 h codeposition experiment with a flux ratio C_2_H_2_:H_2_S:H = 1:1:20 (experiment 1). In the same panel, a control blank experiment of a C_2_H_2_ + H (1:10, experiment 6) deposition is shown in gray. All depositions were performed at 10 K. In comparison with the blank experiment, the IR spectrum of C_2_H_2_ + H_2_S + H contains three additional features at ∼1272, ∼1450, and ∼1591 cm^–1^ due to newly formed reaction products. The bands at ∼1272 and ∼1450 cm^–1^, respectively, are consistent with the peak positions and relative intensities of the CH_2_ wagging and CH_3_ bending modes of CH_3_CH_2_SH, as shown in Figure 4b by the standard spectrum in green (see also Smith et al.^101^). The band at ∼1591 cm^–1^ is compatible with the C=C stretching mode of CH_2_CHSH, its strongest feature.^102^

Infrared spectra are also obtained during the TPD experiments, which allows one to correlate peaks in mass signals with the disappearance of a molecule’s vibrational modes. The difference spectrum between temperatures of 100 and 140 K of the codeposition with C_2_H_2_:H_2_S:H = 1:1:20 (experiment 1) is shown in red in Figure 4b. This is obtained by subtracting the spectrum measured at 140 K from that measured at 100 K, thus highlighting the vibrational modes that disappear during this temperature range. At 100 K, C_2_H_2_ and its hydrogenation products C_2_H_4_ and C_2_H_6_ are already desorbed from the ice, and only the bands of CH_2_CHSH and CH_3_CH_2_SH are visible in the difference spectrum. These bands completely disappear from the spectra between 100 and 140 K. These desorption temperatures obtained from the IR data are consistent with the QMS measurements shown in Figure 3. During warmup, the areas of the bands remain constant until reaching the temperature in which they begin to desorb, indicating that no significant change in their concentration in the ice occurs upon heating. The combination of both the infrared and the QMS data provides unambiguous evidence for the identification of CH_2_CHSH and CH_3_CH_2_SH and signals that these molecules must be formed in the ice at 10 K and remain preserved until desorption.

### H_2_S_2_ and HSCH_2_CH_2_SH

**Figure 5:**
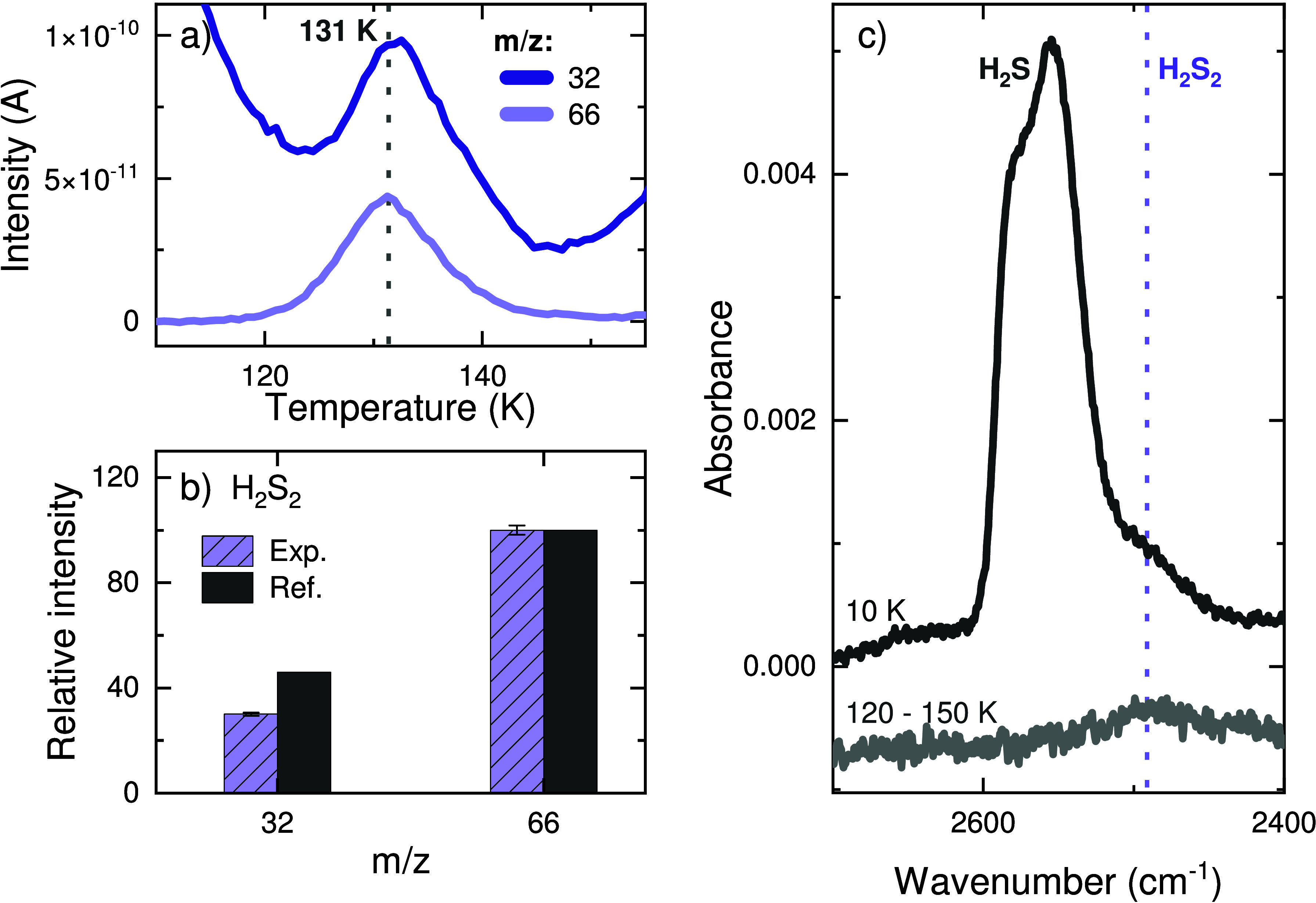
Assignment of the TPD-QMS peak at ∼131 K as disulfane (H_2_S_2_) after a codeposition experiment with a flux ratio of C_2_H_2_:H_2_S:H = 1:1:20 (experiment 1). (a) QMS signals of the relevant fragments. (b) Mass fragmentation pattern corrected for the sensitivity of the QMS in comparison with the standard for H_2_S_2_ from Santos et al.^50^ (c) Infrared spectrum at 10 K (black) and difference spectrum between 120 and 150 K (gray).

The second strongest desorption feature in Figure 1 peaks at 131 K and contains the mass signals *m*/*z* = 32 and 66 (see Figure 5a). Both the peak desorption temperature and the relative intensities of the fragments are in full agreement with the reference values for disulfane (H_2_S_2_)^50^ (Figure 5b). Moreover, its characteristic SH stretching band is observed on the red wing of the H_2_S feature peaking at ∼2491 cm^–1^, as shown in Figure 5c. During TPD, the intensity of the H_2_S_2_ infrared feature remains constant until its complete desorption before 150 K, consistent with the desorption temperature observed for its mass fragments in the QMS data. This is highlighted by the difference spectrum between 120 and 150 K shown in gray in Figure 5c. Indeed, the formation of this species is unsurprising, as it has been previously shown to form at 10 K in an H_2_S ice exposed to H atoms likely as a result of SH radical recombination.^50^

**Figure 6:**
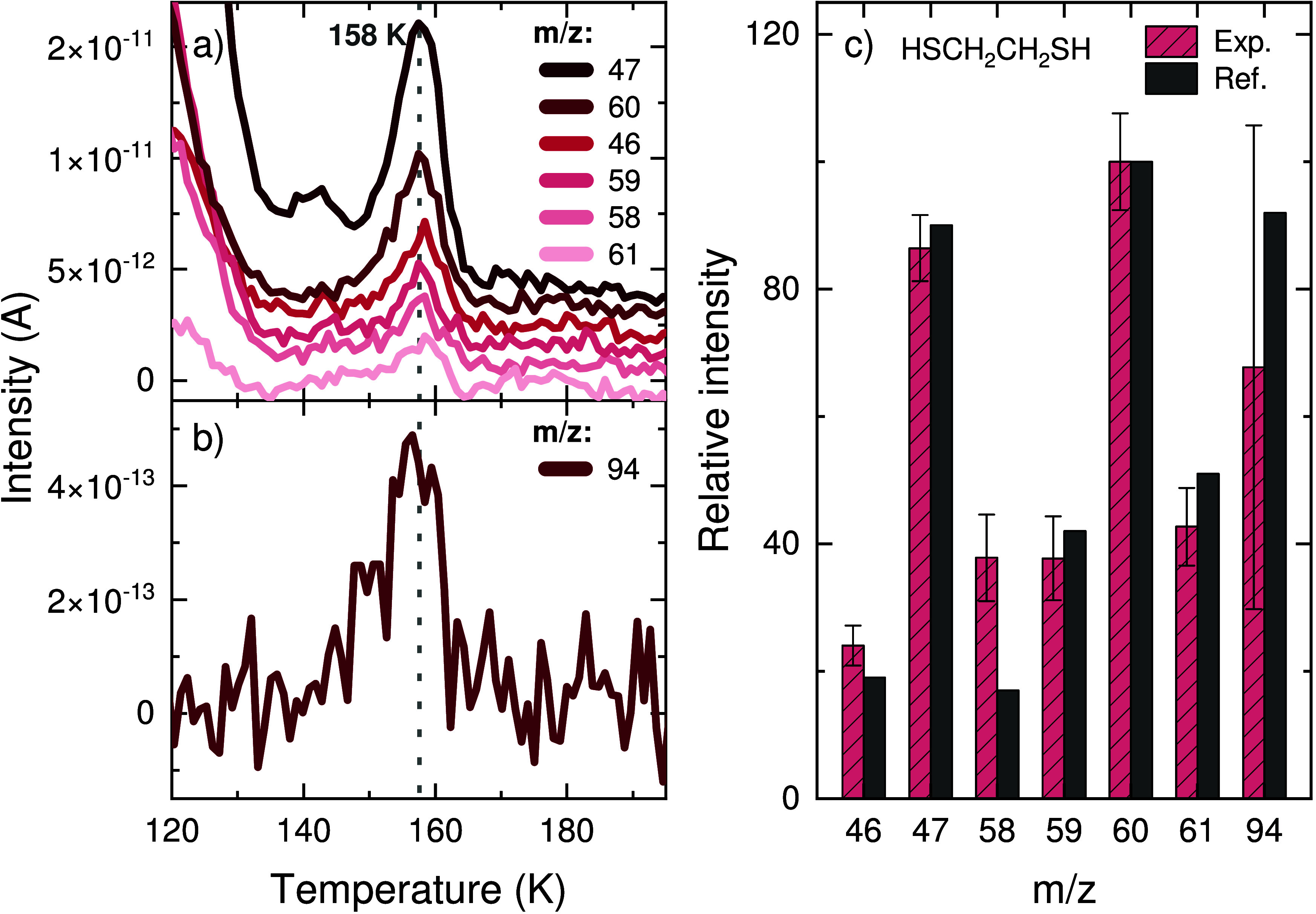
Assignment of the TPD-QMS peak at ∼158 K as 1,2-ethanedithiol (HSCH_2_CH_2_SH) after a codeposition experiment with a flux ratio of C_2_H_2_:H_2_S:H = 1:1:20 (experiment 1). (a) QMS signals of the relevant fragments with *m*/*z* ≤ 61. (b) Signal for *m*/*z* = 94 measured during the same experiment. The dotted gray line indicates the peak desorption temperature. (c) Mass fragmentation pattern of the detected signals for the peak at ∼158 K corrected for the sensitivity of the QMS in comparison with the standard for HSCH_2_CH_2_SH from the NIST. The larger error bar of *m*/*z* = 94 is a consequence of the significantly lower sensitivity of the QMS at this mass-to-charge ratio.

Subsequently to H_2_S_2_, a desorption feature appears at 158 K with contributions from *m*/*z* = 46, 47, 58, 59, 60, 61, and 94 (Figure 6, left panels). The mass-to-charge ratios of 92, 118, 120, and 122 were also recorded during the TPD experiment, but no increase in their signal was observed above the instrumental detection limit. Thus, it is reasonable to assume that *m*/*z* = 94 corresponds to the product’s molecular ion defined by the general formula C_2_H_6_S_2_. Among the possible structures associated with this formula, the most promising candidate for assignment of the band at 158 K is 1,2-ethanedithiol (HSCH_2_CH_2_SH). The relative intensities of the mass fragments match well with the reference values for HSCH_2_CH_2_SH provided by the NIST (Figure 6, right panel), and the desorption temperature is also in line with a slightly higher value of 180 K measured by Roe and Schulz^103^ for one layer of HSCH_2_CH_2_SH on a molybdenum (110) surface with adsorbed carbon.

No corresponding features of HSCH_2_CH_2_SH are observed in the infrared spectra after deposition or during TPD, likely due to the significantly lower sensitivity of the infrared spectrometer in comparison to the QMS employed in our experiments. Moreover, this product’s yield is relatively small, and its IR features heavily overlap with the other, more abundant S-bearing species sharing the same functional groups. Nonetheles, as will be discussed in detail in Computational Results and Chemical Network, the most probable route to form 1,2-ethanedithiol requires the consumption of a CH_2_CHSH molecule as reactant. The fact that the infrared absorbance area of CH_2_CHSH remains constant during TPD until its desorption indicates that no additional reactions involving this molecule take place as a result of the heat, thus suggesting that HSCH_2_CH_2_SH formation should occur at 10 K.

### CH_2_CS and CH_3_CHS

**Figure 7:**
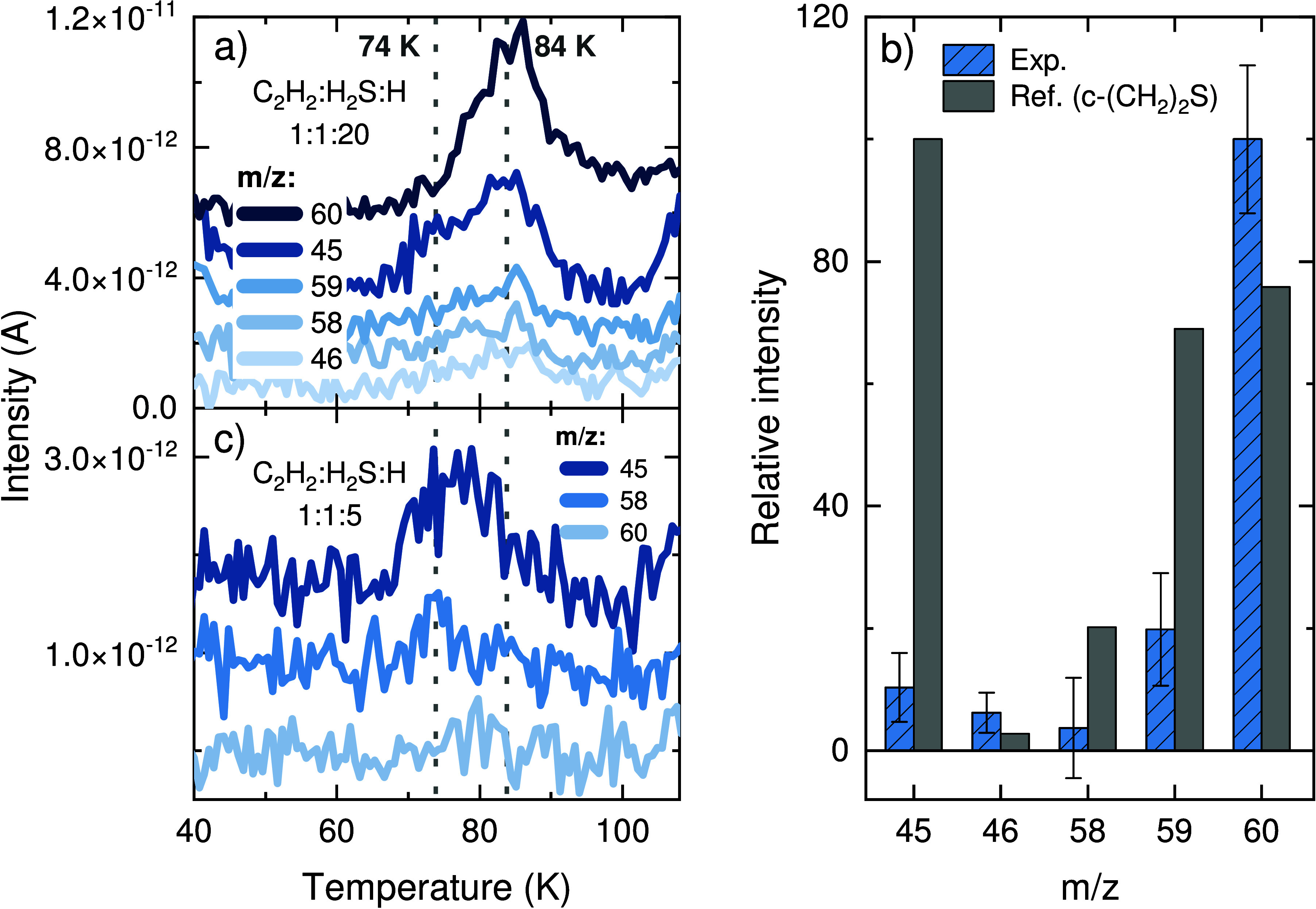
Tentative assignments of the TPD-QMS peaks at ∼74 and ∼84 K as, respectively, thioketene (CH_2_CS) and thioacetaldehyde (CH_3_CHS). (a) QMS signals of the relevant fragments after a codeposition experiment with a flux ratio of C_2_H_2_:H_2_S:H = 1:1:20 (experiment 1). The dotted gray lines indicates the peak desorption temperatures. (b) Mass fragmentation pattern of the detected signals for the peak at ∼84 K corrected for the sensitivity of the QMS (blue), assigned to CH_3_CHS, in comparison to the standard fragmentation pattern of c-(CH_2_)_2_S from the NIST (gray). (c) QMS signals of the relevant fragments after a codeposition experiment with a flux ratio of C_2_H_2_:H_2_S:H = 1:1:5 (experiment 3), in which the CH_3_CHS production is minimized. The dotted line highlights the contribution from the desorption peak at ∼74 K to the signals of *m*/*z* = 58 and *m*/*z* = 45.

Prior to the sublimation of the most abundant products, a relatively small band appears with peak desorption temperature of ∼84 K (see Figure 7a). It is characterized by *m*/*z* = 45, 46, 58, 59, and 60, but without a contribution from higher masses. Because its desorption temperature coincides with H_2_S, we cannot probe the yield of *m*/*z* = 32 from this species. It is reasonable to assume *m*/*z* = 60 to be its molecular ion, described by the chemical formula C_2_H_4_S (i.e., an isomer of vinyl mercaptan). Besides CH_2_CHSH, three other closed-shell structures can derive from this formula: thioacetaldehyde (CH_3_CHS), thiirane (c-(CH_2_)_2_S), and thione S-methylide (CH_2_SCH_2_). The last species is significantly less stable than the other isomers^104^ and would require a cleavage of the C≡C bond in a C_2_H_2_ molecule. Thus, it is unlikely to be synthesized under our experimental conditions. Thiirane is also ruled out due to the incompatibilities of its standard fragmentation pattern from the NIST and the relative intensities of the recorded mass signals (Figure 7b)—in particular considering the large discrepancy in the dominant mass fragment between the two (i.e., *m*/*z* = 45 for thiirane, compared to *m*/*z* = 60 for our experiments). This leaves CH_3_CHS as the most promising candidate for the desorption band at ∼84 K. To the best of our knowledge, no standard mass fragmentation patterns are available for this species in the literature—likely because of its extreme reactivity at room temperature. The fragments detected by QMS are nevertheless all reasonable to arise upon electron-impact ionization of thioacetaldehyde. These are [CHS]^+^ (*m*/*z* = 45), [CH_2_S]^+^ (*m*/*z* = 46), [C_2_H_2_S]^+^ (*m*/*z* = 58), [C_2_H_3_S]^+^ (*m*/*z* = 59), and [C_2_H_4_S]^+^ (*m*/*z* = 60). Given the lack of standard measurements, this assignment is classified as tentative. Similarly to HSCH_2_CH_2_SH, the formation of CH_3_CHS also requires CH_2_CHSH as a reactant (see Computational Results and Chemical Network for more details), and consequently, it is most likely formed at 10 K.

At ∼74 K, a small shoulder appears in the signal of *m*/*z* = 45 (Figure 7a), suggesting the desorption of another species containing the [CHS^+^] moiety. Its identification is, however, quite challenging given the very small yield of this product and its proximity to the more abundant CH_3_CHS. For an ice mixture with mixing ratio C_2_H_2_:H_2_S:H = 1:1:5 deposited for 1 h (experiment 3), the production of CH_3_CHS can be significantly reduced with respect to the shoulder peak at ∼74 K (see Figure 7c), revealing an otherwise undetectable contribution from *m*/*z* = 58 to the fragmentation of the more volatile product. This mass fragment is consistent with the formula C_2_H_2_S^+^ and, together with *m*/*z* = 45, suggests that the peak is due to thioketene (CH_2_CS). This is, however, only a tentative assignment, as a more secure identification is alas not possible given the lack of literature standards on thioketene—presumably due to its high reactivity—and the difficulty in distinguishing this product from the more abundant peak at ∼84 K.

### Computational Results and Chemical Network

**Figure 8:**
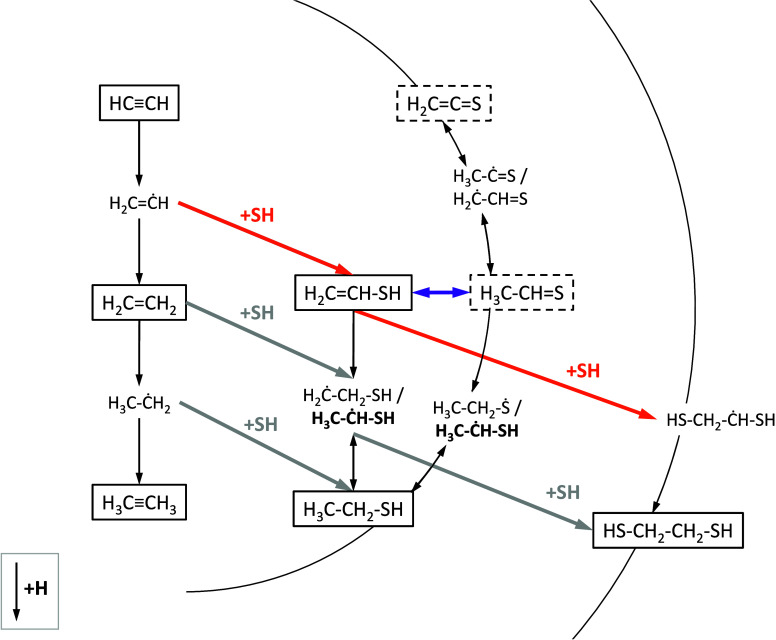
Chemical network explored in this work. Boxes denote closed-shell species with solid and dashed lines indicating confirmed and tentative detections, respectively. Open-shell species in boldface are formed barrierlessly.^105^ Reactions with SH radicals constrained in this work are indicated by orange arrows, gray arrows display potential reactions not investigated further in this work, and black arrows represent reactions with H atoms.The purple arrow highlights the speculated intermolecular isomerization process.

We combine theoretical calculations performed in-house and from the literature to constrain the reactions at play in our experiments. In both cases, care should be taken when interpreting the activation energies since they are obtained in vacuum and therefore could differ from more representative solid-state scenarios. Nonetheless, these values are still useful for an assessment of the feasibility of a given route. The proposed chemical network is summarized in Figure 8. The first step in forming sulfur-bearing species under our experimental conditions involves the association of SH to a hydrocarbon containing two C atoms (general formula C_2_H_*x*_). The radical-molecule reaction between C_2_H_2_ and SH is the most straightforward candidate to initiate the network:
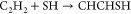
6

Our DFT calculations in vacuum predict a fairly small energy barrier of only Δ*E*_a_ ≈ 758 K for this reaction. However, due to the extremely low temperatures of our experiments (10 K) and the high mass of the SH radical, quantum tunneling is largely restricted. Considering that reaction 2 is exothermic, vibrationally excited SH radicals might play a role in overcoming such a barrier. However, energy dissipation in H_2_O ices takes place typically within picosecond time scales (e.g., Ferrero et al.^106^), and we extrapolate that it will also proceed in similarly short time scales for H_2_S mixed with C_2_H_2_, since a variety of vibrational modes are present that could accept the energy that is dissipating. Thus, reaction 6 is unlikely to initiate the chemical network probed here.

Alternatively, the network is proposed to be kicked off by the formation of C_2_H_3_ through a hydrogen addition reaction to C_2_H_2_ and its subsequent association with SH:
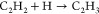
7
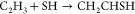
8

Reaction 7 has been previously investigated in experimental as well as theoretical works and has been shown to proceed efficiently on icy surfaces at ∼10 K,^74−76,107^ with a predicted activation energy of ∼2141–2430 K depending on the level of theory utilized.^76^ Despite being significantly larger than the activation energy for reaction 6, it does not hinder reaction 7 due to the much more efficient quantum tunneling of the light H atoms as opposed to the heavy SH radicals. Indeed, estimated rate constants for reaction 7 in the solid phase are of the order of 10^4^ s^–1^.^76^ The subsequent reaction 8 takes place between two radicals, C_2_H_3_ and SH. The former is a nonpolar species and hence is expected to have a low binding energy to the H_2_S ice. Since the ice is continuously grown, a randomized distribution of orientations is to be expected. Thus, reorientation barriers as predicted by Enrique-Romero et al.^108^ and observed by, e.g., Martín-Doménech et al.^109^ are less of a limitation. Consequently, nondiffusive interactions between C_2_H_3_ and SH radicals in the vicinity of one another are expected to efficiently form CH_2_CHSH and initiate the sulfur chemical network in Figure 8. Such nondiffusive mechanisms have been extensively studied in the laboratory and through chemical modeling and are suggested to contribute significantly to ice chemistry under dark cloud conditions (Jin and Garrod,^110^ Garrod et al.,^111^ and references therein).

Following its formation via reaction 8, CH_2_CHSH can be hydrogenated to form CH_3_CH_2_SH:
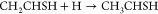
9a
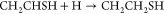
9b
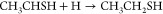
10a
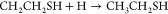
10bwhich proceeds by first forming either the radical CH_3_CHSH (reaction 9a, Δ*E*_a_ = 0 in vacuum) or CH_2_CH_2_SH (reaction 9b, Δ*E*_a_ ≈ 1227 K in vacuum).^105^ The subsequent hydrogen additions in reactions 10a and 10b proceed without an activation barrier.

Alternatively, CH_2_CHSH can also react with nearby SH radicals to form the doubly sulfurated radical HSCH_2_CHSH:
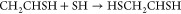
11for which no activation energy barrier was observed in our vacuum DFT calculations. The hydrogenation of this radical will lead to the formation of HSCH_2_CH_2_SH:
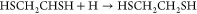
12

Similarly, radical–radical coupling reactions between SH + CH_2_CH_2_SH/CH_3_CHSH could also form HSCH_2_CH_2_SH efficiently.

A third possible fate for the CH_2_CHSH molecule is that it is converted to its structural isomer CH_3_CHS:
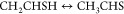
13

Intramolecular isomerization (i.e., a transfer of a hydrogen atom within one molecule) is unlikely to occur at 10 K given its high energy barrier (Δ*E*_a_ ≳ 28000 K in vacuum).^104,105^ Alternatively, transfers of hydrogen atoms in a concerted mechanism involving multiple molecules from the ice are associated with significantly lower activation energies and could arguably occur in our experiments. This process, known as “intermolecular isomerization”, has been widely studied in both the gas and liquid phases for the oxygen-bearing counterparts CH_2_CHOH and CH_3_CHO^112−115^ and is shown to be catalyzed by surrounding H_2_O molecules.^113,114^ This same mechanism has been proposed previously to explain the large abundances of CH_2_CHOH and CH_3_CHO in similar ice experiments involving C_2_H_2_ and OH radicals.^19^ However, Perrero et al.^22^ found this mechanism to be hampered by high activation energy barriers (≳7000 K) in pathways starting from both CH_2_CHOH and its radical precursor CH_2_CHO. Interestingly, theoretical calculations by Suenobu et al.^116^ predict a faster conversion of CH_2_CHSH into CH_3_CHS mediated by H_2_O molecules in the liquid phase compared to the oxygen-bearing counterparts. They argue that this is due to the larger electric dipole moment of the transition state in the sulfur-bearing case, causing it to be more stabilized in aqueous environments. We therefore speculate that such a mechanism could also proceed in the solid state under our experimental conditions facilitated by H_2_S molecules in the vicinity of CH_2_CHSH, although further theoretical work is warranted to accurately constrain this possibility.

We emphasize that CH_3_CHS is tentatively detected in this work and contains relatively low abundances. Thus, its suggested formation by the intermolecular isomerization mechanism is probably not very efficient. Nonetheless, assuming that it can be formed, further hydrogenation will lead to CH_3_CH_2_SH via
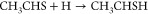
14a
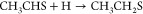
14b
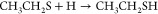
15

Reaction 14a proceeds barrierlessly, whereas reaction 14b has a very small Δ*E*_a_ of ∼397 K.^105^ Both CH_3_CHSH and CH_3_CH_2_S will be hydrogenated without an activation energy through reactions 10a and 15 to form CH_3_CH_2_SH. Finally, CH_3_CHS can potentially undergo two hydrogen-abstraction reactions to form CH_2_CS, another tentative product of this work. This alluded abstraction route warrants dedicated experimental and theoretical investigations to be confirmed, as only tentative detections of both reactants and products are provided here.

Abstraction reactions from the radicals CH_2_CH_2_SH and CH_3_CHSH are also possible a priori and are explored computationally here. Our DFT calculations predict that CH_2_CH_2_SH can undergo abstraction routes induced by both H atoms and SH to form thiirane (c-(CH_2_)_2_S):
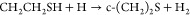
16
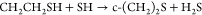
17

Likewise, CH_3_CHSH can also form thiirane by reacting with H:
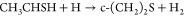
18

This structure, however, is excluded as a major product in our experiment on the basis of its mass fragmentation pattern (see CH2CS and CH3CHS). The reason behind this discrepancy between theory and experiment may be related to the former being performed in vacuum, when interactions with other species in the ice could potentially hinder the formation of c-(CH_2_)_2_S.

Most of the hydrogenation steps in this network ultimately lead to the formation of CH_3_CH_2_SH. This molecule could in principle be further processed by reactions with H atoms to form the radicals CH_3_CH_2_S, CH_3_CH_2_, CH_3_CHSH, or CH_2_CH_2_SH.^117^ The theoretical calculations by Zhang et al.^117^ predict that the hydrogen abstraction channel from the SH group (CH_3_CH_2_SH + H → CH_3_CH_2_S + H_2_) is the dominant route in vacuum (Δ*E*_a_ ≈ 1610 K), but that the C–S bond breaking channel (CH_3_CH_2_SH + H → CH_3_CH_2_ + H_2_S) is also likely to proceed (Δ*E*_a_ ≈ 1761 K). The contribution from these channels might vary when surfaces are considered (as was shown by Nguyen et al.^118^ within their work and in comparison to Lamberts^119^ for the analogous reactions involving CH_3_SH). We do not find any evidence (such as a peak in mass signal) for the presence of CH_3_CH_2_SSCH_2_CH_3_ (diethyl disulfide, DEDS) in our experiments—the presumed product of the recombination of two CH_3_CH_2_S radicals. This is rather unsurprising, since the addition routes to CH_3_CH_2_SH proceed effectively barrierlessly and multiple reaction steps, as well as close proximity of reactants, would be needed to form DEDS. Overall, the chemical network probed here converges into forming mostly CH_3_CH_2_SH as long as enough H is available. This conclusion can assist in explaining astronomical observations of S-bearing COMs with two carbon atoms (or lack thereof), as will be discussed in Astrophysical Implications.

## Astrophysical Implications

Within interstellar clouds, low activation barriers are generally required for a chemical reaction to occur, as thermal hopping is not possible for most molecular radicals at the typical temperatures of those environments (10–20 K). The high reactivity associated with open-shell species thus makes radicals important drivers of chemical complexity in such astronomical environments. This is particularly relevant for solid-state reactions facilitated by interstellar dust grains. In the case of sulfur, the SH radical can serve as an important pivot toward building a complex sulfur-bearing inventory, especially because it is expected to be continuously formed in interstellar ices throughout a wide evolutionary span. During the earlier cloud stages, it can be readily produced by the hydrogenation of sulfur atoms accreted onto interstellar dust grains. As the density of the environment increases, most of the atomic sulfur is expected to be readily converted into H_2_S. Abstraction reactions involving H atoms and H_2_S can then efficiently reform SH, thus partially replenishing the supply of this radical in the ice.

The deuterium fractionation of H_2_S observed in Class 0 sources points to an early formation in ices, before the CO catastrophic freeze-out stage.^120^ Similarly, C_2_H_2_ is expected to be more abundantly present in earlier cloud stages, where atomic carbon is available to produce C_2_H_2_ via the bottom-up route, and before C_2_H_2_ is largely hydrogenated to form C_2_H_4_ and C_2_H_6_. Reactions involving the two species and the ubiquitous hydrogen atoms are, therefore, feasible given that they coexist in the same ice environment. As illustrated in Figure 8, these interactions can enrich interstellar ices with complex organic sulfur-bearing molecules. Furthermore, C_2_H radicals could act as an additional source of carbon within our network upon adsorption onto ice grains. It should be noted, however, that the scheme proposed here could be further complicated in fully representative interstellar ices due to the presence of other species, in particular water.

**Figure 9:**
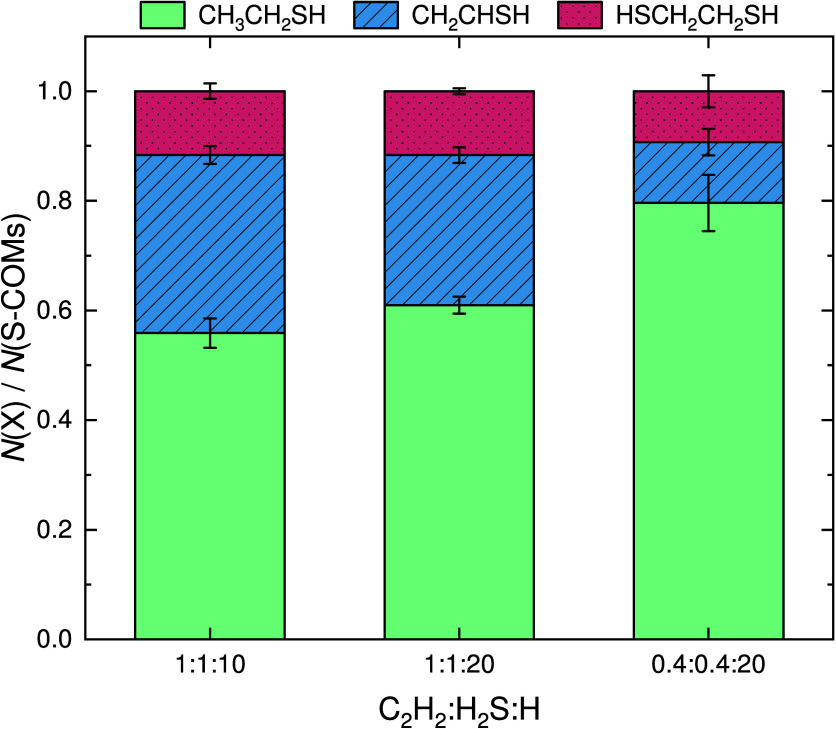
Relative abundances of the sulfur-bearing COMs formed during deposition as a function of the C_2_H_2_:H_2_S:H ratio. The abundances are shown with respect to the total yield of sulfurated COMs positively identified at the end of each experiment.

For the three main organic products, CH_3_CH_2_SH, CH_2_CHSH, and HSCH_2_CH_2_SH, the dependence of the product yield with the molecule-to-hydrogen ratio is shown in Figure 9. The products’ relative column densities are derived from the mass signals of their molecular ions using eq 4 and the parameters in Table 1. Thioacetaldehyde and thioketene (CH_3_CHS and CH_2_CS, respectively) are not included in this analysis since they are tentative detections and minor products. For all explored deposition conditions, the most abundant S-bearing organic molecule resulting from the interaction of C_2_H_2_ with H_2_S and H atoms is CH_3_CH_2_SH. The predominance of this species is in line with the conclusions drawn by Shingledecker et al.^105^ from their calculations and the computational results from this work. Once C_2_H_2_ is hydrogenated to form C_2_H_3_, it can react barrierlessly with SH radicals to form CH_2_CHSH, which can subsequently be barrierlessly hydrogenated to form CH_3_CH_2_SH. The production of CH_3_CH_2_SH can therefore proceed very efficiently as long as there are H atoms in the vicinity available to react and hence is increasingly favored for higher H-to-molecule ratios. Thus, our experiments show that CH_3_CH_2_SH acts as a sink in the chemical network explored here, meaning that the sulfur budget in this network will be mostly locked away into ethanethiol at the expense of the other products—in agreement with the theoretical predictions.^105^ These conclusions remain valid irrespective of the formation route behind CH_2_CHSH, and thus are not exclusively dependent on the network being initiated by the interaction between C_2_H_3_ and SH. Indeed, as suggested by Shingledecker et al.,^105^ the absence of CH_2_CHSH in interstellar sources despite dedicated attempts to identify it toward Sgr B2(N2),^121^ alongside the detections of CH_3_CH_2_SH toward both Orion KL and the G+0.693–0.027 molecular cloud,^122,123^ might be related to the effective chemical conversion of the former to the latter.

Thioketene (CH_2_CS) was also detected in interstellar environments, toward the cold core TMC-1.^124^ The reaction routes proposed in this work could, in principle, contribute to its formation in the solid phase, albeit to a small extent. Nonetheless, its astronomical detection reinforces the relevance of investigating chemical networks that lead to sulfur-bearing organics with two carbon atoms.

## Conclusions

In the present work, we explore the solid-state chemistry resulting from the interaction of C_2_H_2_, H_2_S, and H atoms in interstellar ice at 10 K by means of laboratory experiments combined with theoretical calculations. The investigated chemical network is summarized in Figure 8, and our main findings are listed below.

(1) The codeposition of C_2_H_2_, H_2_S, and H leads to the formation of several sulfur-bearing species at 10 K via radical-induced reactions involving SH. We securely identify the products CH_3_CH_2_SH, CH_2_CHSH, HSCH_2_CH_2_SH, H_2_S_2_, and tentatively CH_3_CHS and CH_2_CS, by using infrared spectroscopy and mass spectrometry.
(2) Calculations at the M062X/def2-TZVP level of theory bechmarked with CCSD(T)-F12/AUG-CC-PVTZ predict an activation barrier of ∼758 K for the radical-molecule reaction C_2_H_2_ + SH → C_2_H_2_SH, which is deterred under molecular clouds conditions. Thus, the chemical network is likely initiated by the interaction between C_2_H_3_ and SH radicals.
(3) The product CH_2_CHSH plays an important role as an intermediate, as it can be hydrogenated to form CH_3_CH_2_SH or potentially converted to its isomer CH_3_CHS. Given enough H atom availability, it will be largely consumed to produce more stable species.
(4) For all explored deposition conditions, the main product formed is CH_3_CH_2_SH, with percentage yield with respect to the sum of S-bearing COMs ranging from ∼56% to ∼80%. The yield of CH_3_CH_2_SH increases with the H fraction due to its efficient formation through a series of barrierless hydrogenation reactions. It therefore acts as a sulfur sink in the present chemical network, being preferably formed at the expense of the other products.

Astronomical detections of CH_3_CH_2_SH and CH_2_CS in the submillimeter range evince the importance of exploring the formation mechanisms of sulfur-bearing organic molecules with two carbon atoms under interstellar cloud conditions. In this work we experimentally investigate solid-phase formation routes that enrich the ice with the aforementioned products, in particular during the early cloud stages. Nonetheless, further laboratory, observational, and modeling works are warranted to better constrain the ice abundances of C_2_H_2_ and H_2_S and the yield of products in more representative interstellar ices.
